# Draft Genome Sequence of *Vibrio* (*Listonella*) *anguillarum* ATCC 14181

**DOI:** 10.1128/genomeA.01185-16

**Published:** 2016-10-20

**Authors:** Christopher J. Grim, Harold J. Schreier

**Affiliations:** a Center for Food Safety and Applied Nutrition, Office of Applied Research and Safety Assessment, U.S. Food and Drug Administration, Laurel, Maryland, USA; b Department of Marine Biotechnology, Institute of Marine and Environmental Technology, University of Maryland Baltimore County, Baltimore, Maryland, USA; c Department of Biological Sciences, University of Maryland Baltimore County, Baltimore, Maryland, USA

## Abstract

We report the draft genome sequence of *Vibrio anguillarum* ATCC 14181, a Gram-negative, hemolytic, O2 serotype marine bacterium that causes mortality in mariculture species. The availability of this genome sequence will add to our knowledge of diversity and virulence mechanisms of *Vibrio anguillarum* as well as other pathogenic *Vibrio* spp.

## GENOME ANNOUNCEMENT

*Vibrio anguillarum* is a causative agent of vibriosis, affecting cultured and wild finfish and shellfish in marine, brackish, and fresh water environments (1). While more than 20 serotypes have been identified for this species, serotypes O1 and O2 are primarily responsible for vibriosis disease (2, 3). Here, we announce the genome sequence of the O2 serotype, α-hemolytic, *V. anguillarum* strain ATCC 14181 (Bergeman, NCMB 829), to facilitate identification of factors and processes involved in pathogenesis and to add to our understanding of the diversity and evolution of this species.

A single colony was grown in marine broth 2216 (Difco) at 28°C, and DNA was extracted with the Wizard genomic DNA purification kit (Promega). Genomic insert libraries were prepared using the Nextera XT DNA library prep kit (Illumina) and sequenced with a MiSeq (Illumina) benchtop sequencer, using 2 × 250-bp paired-end V2 chemistry. The read library contained 2,396,116 trimmed paired-end reads with an average read length of 228 bp and an average coverage of 135×. *De novo* assembly of the paired-reads was performed using the CLC Genomics Workbench assembly tool (CLC Bio/Qiagen), yielding 217 contigs with an average length of 18,502 bp and a total genome size of 4,014,900 bp. The *N*_50_ of this assembly is 57,813 bp with a G+C composition of 44.5%. Gene prediction and annotation using the RAST (Rapid Annotations using Subsystems Technology) server (4, 5) generated 3,672 protein-encoding genes and 75 total RNAs.

The genome contains quorum-sensing *luxT, luxR,* and *hapR* regulators, and autoinducer 1 and 2 system components. Genes encoding type IV pili, flagellar (*flgF*), and motility (*motY*) structures that are utilized for biofilm production (6), as well as gene clusters associated with capsule and exopolysaccharide production (*cps*, *vps*, and *eps*) (6), were identified.

Genes encoding the virulence-associated RTX toxin and accompanying binding and transport were found, a characteristic shared by O1 serotype strains 775 and 96 F (7), but not by O2 serotype strain RV22, which lacks the RTX toxin (7). Elements for at least one putative integrase-containing prophage genome were identified using PHAST (8). The hemolysin-related protein RbmC (9) and at least seven putative hemolysins, including a thermolabile hemolysin and the virulence-related pore-forming HlyA (6), were found, as well as the *toxRS* virulence regulator and genes encoding types I and VI secretion system components. Iron acquisition components, TonB (10) and *fhu* ferrichrome transport genes (11), and vibriobactin and yersiniabactin-like ferric transport systems were identified. Proteases important for *Vibrio* pathogenicity (12, 13) included metalloproteases, collagenases, and three vibriolysins. In addition, the genome harbors six chitinase-encoding genes.

The genome also encodes 931 hypothetical proteins with no significant similarity to any protein in GenBank (25% of the open reading frames). Comparisons of these, as well as pathways defined by the genes noted above, with genomes from other *V. anguillarum* and related *Vibrio* spp. will provide further insight into their pathogenicity and evolution.

### Accession number(s).

This whole-genome shotgun project has been deposited at DDBJ/ENA/GenBank under the accession number MCJC00000000. The version described in this paper is the first version, MCJC01000000.
